# Reliability and concurrent validity of a novel method allowing for in-shoe measurement of navicular drop

**DOI:** 10.1186/1757-1146-7-12

**Published:** 2014-02-13

**Authors:** Birgitte H Christensen, Kathrine S Andersen, Kristina S Pedersen, Britt S Bengtsen, Ole Simonsen, Simon L Kappel, Michael S Rathleff

**Affiliations:** 1 Orthopaedic Surgery Research Unit, Aalborg University Hospital, Aalborg, Denmark; 2 Department of Occupational and Physiotherapy, Aalborg University Hospital, Aalborg, Denmark; 3 Department of Health Science and Technology, Aalborg University, Aalborg, Denmark; 4 Signal processing and control group, Aarhus School of Engineering, Aarhus University, Aarhus, Denmark

**Keywords:** Navicular drop, Foot kinematics, In-shoe, Stretch-sensor

## Abstract

**Background:**

Increased navicular drop is associated with increased risk of lower extremity overuse injuries and foot orthoses are often prescribed to reduce navicular drop. For laboratory studies, transparent shoes may be used to monitor the effect of orthoses but no clinically feasible methods exist. We have developed a stretch-sensor that allows for in-shoe measurement of navicular drop but the reliability and validity is unknown. The purpose of this study was to investigate: 1) the reliability of the stretch-sensor for measuring navicular drop, and 2) the concurrent validity of the stretch-sensor compared to the static navicular drop test.

**Methods:**

Intra- and inter-rater reliability was tested on 27 participants walking on a treadmill on two separate days. The stretch-sensor was positioned 20 mm posterior to the tip of the medial malleolus and 20 mm posterior to the navicular tuberosity. The participants walked six minutes on the treadmill before navicular drop was measured. Reliability was quantified by the Intraclass Correlation Coefficient (ICC 2.1) and agreement was quantified by Limits of Agreement (LOA). To assess concurrent validity, static navicular drop was measured with the stretch-sensor and compared with static navicular drop measured with a ruler on 27 new participants. Linear regression was used to measure concurrent validity.

**Results:**

The reliability of the stretch-sensor was acceptable for barefoot measurement (intra- and inter-rater ICC: 0.76-0.84) but lower for in-shoe measurement (ICC: 0.65). There was a significant association between static navicular drop measured with the stretch-sensor compared with a ruler (r = 0.745, p < 0.001).

**Conclusion:**

This study suggests that the stretch-sensor has acceptable reliability for dynamic barefoot measurement of navicular drop. Furthermore, the stretch-sensor shows concurrent validity compared with the static navicular drop test as performed by Brody. This new simple method may hold promise for both clinical assessment and research but more work is needed before the method can be recommended.

## Background

Reliable information on foot movement is essential for optimal individual prevention and treatment of foot-related disorders. The information acquired in a clinical setting is often restricted to static assessments and visual observations during walking and running. A high degree of foot eversion is associated to lower extremity over-use injuries, why the reliability of the various measurements for foot eversion has been the subject of several studies [1]. Foot eversion involves a complex tri-planar movement in multiple joints. The navicular drop (ND) is a simple and clinically feasible proxy for foot eversion, and describes the range of sagittal deformation of the midfoot. ND has been suggested as the most appropriate parameter for a clinical assessment of foot eversion [2,3] and is a valid indicator of talonavicular motion [4] and rear foot movement [5]. Previous research on the risk of developing injury has highlighted the importance of ND, as an increased ND is associated with increased risk of overuse injuries, such as medial tibial stress syndrome and patellofemoral pain syndrome [6,7].

Foot orthoses are often prescribed for, and are generally believed to play an important role in, the prevention and treatment of different overuse injuries [8,9]. Franklyn-Miller et al. demonstrated a positive effect of foot orthoses in the prevention of overuse injuries, with an absolute risk reduction of 0.49 [8]. Foot orthoses may potentially reduce eversion by supporting the medial longitudinal arch, which may be measured as a decrease in ND [10,11]. Until now, it has only been possible to measure ND with static assessments, such as the navicular drop test as performed by Brody [12], or during bare foot walking and running, or by using special transparent shoes [13,14]. A simple reliable method to measure ND in conventional shoes is highly desirable. A new stretch-sensor has been developed that allows for in-shoe measurements of ND [15]. However the reliability and validity of the stretch-sensor is unknown. The purpose of this study was to investigate the reliability of the stretch-sensor during barefoot and shod walking and investigate the concurrent validity of the stretch-sensor compared with the static navicular drop test.

We hypothesised that (i) the stretch-sensor would be intra- and inter-rater reliable for measuring barefoot and in-shoe ND (Intraclass Correlation Coefficient > 0.75), and (ii) a high correlation (r > 0.75) between static ND as performed by Brody and static ND measured with the stretch-sensor.

## Methods

The study consisted of two parts. Part 1 was an intra- and inter-rater within- and between-day reliability study and part 2 was a concurrent validity study. The study population was a convenience sample and all participants were recruited from Aalborg University. The inclusion criteria for participants were: no previous or present injury or pain in the lower extremities or back, no medical or neurological conditions, and the ability to walk on a treadmill for a minimum of 20 minutes. Exclusion criteria were: < 18 years of age and BMI > 30. Furthermore participants were not included if they had a highly supinated foot posture and abnormal hypomobility of their midfoot kinematics identified after clinical examination. The study was approved by Aalborg University and conducted in accordance with the Helsinki Declaration and all participants were given written and verbal information about the project and signed an informed consent before participating. The reporting of the study follows the Guidelines for Reporting Reliability and Agreement Studies (GRRAS) [16].

### Description of the stretch-sensor

**Figure 1:**
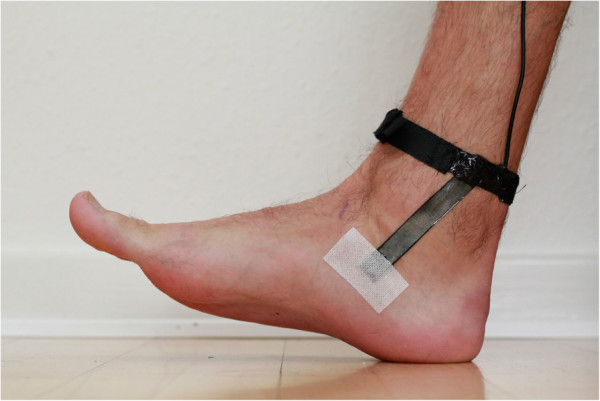
The stretch-sensor is attached on the medial side of the foot between 20 mm posterior to the malleolis medialis, and 20 mm posterior to the navicular tuberosity.

**Figure 2:**
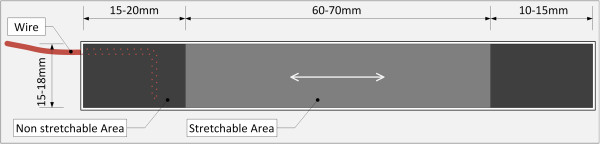
Schematic of the stretch-sensor.

The stretch-sensor is an elastic, flexible, and thin capacitive sensor (Figures 1 and 2). It consists of a stretchable active area that is 15 × 60 mm and a non-stretchable area at both ends that are each 15 × 10 mm, which serve to attach the stretch-sensor to the skin. A change in the stretch of the active area of the sensor causes a linear change in the electrical capacitance. Therefore changes in elongation can be calculated based on the change in the electrical capacitance of the sensor [15]. The thickness of the stretchable area is 0.40–0.60 mm and the thickness is below 1.5 mm in the non-stretchable area, which allows the stretch-sensor to measure in-shoe ND in conventional shoes. The capacitance of the stretch-sensor is measured 200 times per second (200 Hz). The signals from the stretch-sensor are sent to an input box that records the capacitance data on a SD card or transmits the data directly to the computer through a USB cable. Afterwards, the data were analysed using a custom-written script in Matlab [15]. We previously compared the amount of stretch from a calibration slate with the stretch measured from the stretch-sensor and found that the stretch-sensor was valid when compared to a calibration sled with R^2^ = 0.999 [15].

### Attachment of the stretch-sensor to the foot

The stretch-sensor was placed between two points on the medial side of the foot. One attachment point was 20 mm posterior to the malleolus medialis and secured around both malleoli using a Velcro strap which ensured fixation of the sensor. The second attachment point was 20 mm posterior and 20 mm distal to the navicular tuberosity (Figure 1). The choice of attachment points was based on a pilot study in which we investigated different attachment points [15]. The prominence of the malleolus medialis did not allow us to position the distal part of the stretch-sensor directly onto the navicular bone because the stretch-sensor would interfere with the prominence of the malleolus medialis. Therefore, we choose to position it posterior and distal to the navicular tuberosity (Figure 1). Based on previous bone-pin studies by Wolf et al., it appears the entire medial midfoot moves in the same direction during walking [17]. Therefore, the position posterior and distal to the navicular tuberosity is likely a good proxy of ND [15,18]. The attachment of the stretch-sensor took approximately 2 minutes per participant and each rater practiced the placement of the stretch-sensor on a minimum of 20 subjects before placing the stretch-sensor on the participants included in the study.

### Part 1: reliability

Between-day intra-rater, and within- and between-day inter-rater reliability was based on measurements from 27 participants (12 women, 15 men, mean age of 26 years [age 22–39], mean BMI 22.6 [range 19.4–30.0]) recruited from Aalborg University. Rater 1 and rater 2 collected data independently and were blinded to the results from the other rater. In a randomised order, either rater 1 or rater 2 started by positioning the stretch-sensor on the medial side of the foot. Participants then walked without shoes for six minutes on a treadmill [18,19], which was followed by 1.5 minutes of walking that was recorded using the stretch-sensor. The analysis was made on 10 consecutive steps identified after 30 seconds of recording. After this measurement, the stretch-sensor was repositioned by the other rater, and a second recording was made.

**Figure 3:**
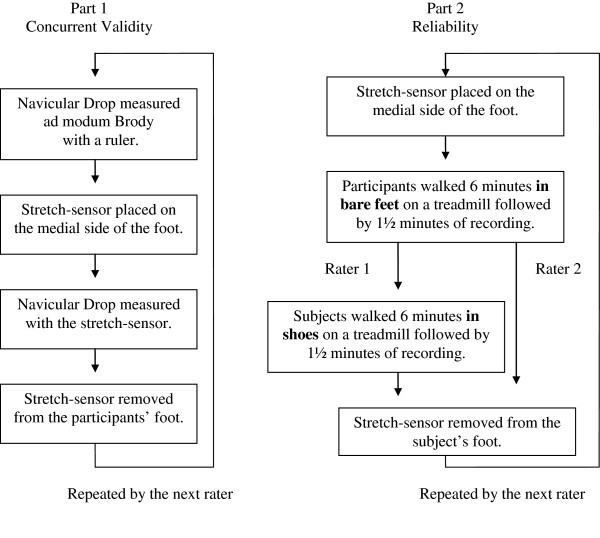
**Flow chart of the three parts of the study.** Part 2 was repeated one day after the first test.

After the barefoot measurements when rater 1 had positioned the stretch-sensor, the procedure was repeated, but this time participants wore shoes while walking on the treadmill (Figure 3). This resulted in two measurements by rater 1 where participants walked with and without shoes while only one measurement was obtained by rater 2, where participants walked without shoes.

The following day, all participants returned and the procedure was repeated. All data were analysed by a third person who was not involved in the data collection.

### Part 2: concurrent validity

To investigate the concurrent validity, the static ND was measured with the stretch-sensor and compared with the static ND as performed by Brody measured simultaneous. ND was defined as the change in the height of the naviculare tuberosity from a neutral position to a relaxed stance [12]. Subtalar neutral position was defined as the position where the talus could be palpated equally on the medial and lateral side of the foot [12]. The ND test as performed by Brody was chosen because it is one of the most commonly used clinical measurements of ND. Static ND was measured on 27 new participant (15 women and 12 men, mean age of 25 years [range 18–28], mean BMI 23.5 [range 19.7-30.0]).

The procedure for the measurement of ND was as follows: Rater 1 measured ND as performed by Brody with a ruler and then placed the stretch-sensor on the medial side of the participant’s foot. This was followed by a measurement of ND recorded by the stretch-sensor. Rater 2 repeated the same procedure, and the stretch-sensor was repositioned between the two procedures (Figure 3). Each rater was blinded to the results of the other rater’s assessments, and a third person registered the measurements of ND from the stretch-sensor, preventing raters 1 and 2 from seeing these results. To test the concurrent validity data from rater 1 was used.

### Data analysis

**Figure 4:**
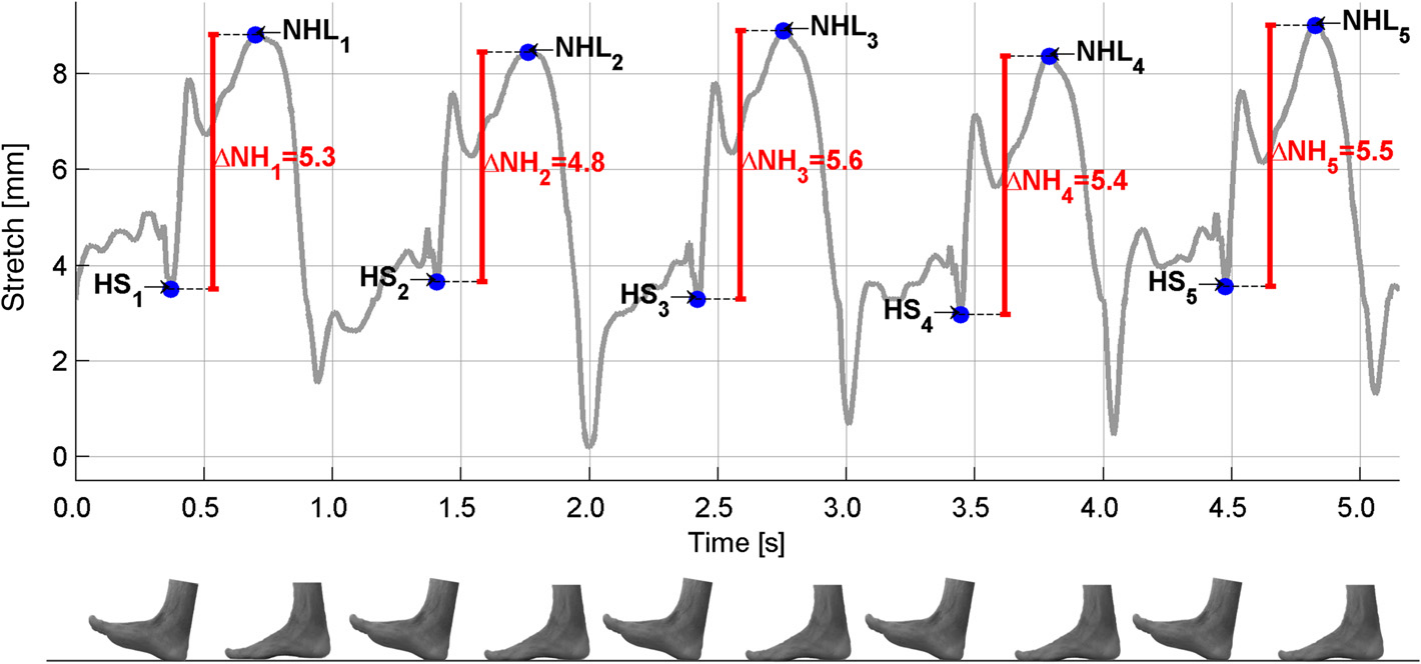
**Measurement of the ND (ΔNH) by the DataAnalyzer.** HS: Heel strike, NHL: Navicular height loaded. ND is described by the difference between NHL and HS.

To calculate ND for part 1 the data from the stretch-sensor was processed in the custom-written Matlab script called DataAnalyzer. Figure 4 shows the calculation of ND during five consecutive steps. Heel strike and the time point where the stretch-sensor was maximally stretched (which corresponds to the minimal height of the navicular during the stance phase), were marked in DataAnalyzer. ND was then described as the difference between the elongation of the stretch-sensor in the two positions. This approach was based on previous studies investigating dynamic ND which also defined ND as the difference in navicular height from heel strike to the minimal height of the navicular during the stance phase [18]. The calculations were based on 10 consecutive steps, which were identified 30 seconds into the recording. The procedure took approximately 3 minutes per participant. In part 2, the data from the stretch-sensor were collected and visualised directly by the program Datalogger, and ND was calculated as the change in the elongation of the stretch-sensor from subtalar neutral to relaxed stance.

### Statistical analysis

In part 1, a two-way random effect model (2.1), single measures, absolute agreement, Intraclass Correlation Coefficients (ICC) were used to express intra- and interrater reliability. ICC > 0.75 was interpreted as acceptable reliability. Limits of Agreements (LoA) were used to express the agreement between the raters [20]. The LoA was calculated as the mean difference between raters ± 1.96 times the standard deviation of the differences between raters. The LoA was presented as a range indicating the maximal potential difference between the two raters in 95% of the ratings. Heteroscedasticity was visually assessed, and there were no trends towards heteroscedasticity. In part 2, a linear regression model was used to investigate the linear association between static ND as measured by the stretch-sensor and static ND as performed by Brody. All the statistical analyses were performed in SPSS 20.0.

## Results

### Part 1: reliability

There were no systematic differences from test to retest except for inter-rater, between-day reliability (Table 1). Reliability ranged from ICC 0.76 (95% CI: 0.53-0.89) to 0.84 (95% CI: 0.68–0.93) when participants were walking without shoes. The reliability when participants were wearing conventional shoes decreased to ICC 0.65 (95% CI: 0.33–0.84) and LoA ranged from -2.4 to 2.6 mm (Table 1).

**Table 1:** The reliability of the stretch-sensor as described by the Intraclass Correlation Coefficient (ICC) and Limits of Agreement (LOA)

**Conditions**	**Mean ND (±SD)**	**Mean difference (95% CI)**	**ICC (95% CI)**	**LOA [mm]**
Intra-rater between-day, rater 1	3.2 (±1.2)	-0.3 (-0.7; 0.0)	0.77 (0.55–0.89)	-2.0; 1.4
Intra-rater between-day, rater 2	3.3 (±1.6)	0.3 (-0.1;0.8)	0.78 (0.52–0.90)	-1.6; 2.1
Inter-rater within-day, day 1	3.2 (±1.4)	-0.2 (-0.6; 0.1)	0.84 (0.68–0.93)	-1.7; 1.3
Inter-rater within-day, day 2	3.6 (±1.6)	-0.2 (-0.6; 0.3)	0.76 (0.56–0.88)	-2.4; 2.0
Inter-rater, between-day (rater 1, day 1 vs. rater 2 day 2)	3.2 (±1.2)	-0.3 (-0.6; 0.1)	0.78 (0.53-0.90)	-2.1; 1.6
Inter-rater, between-day (rater 2, day 1 vs. rater 1 day 2)	3.3 (±1.6)	-0.5 (-0.8; -0.2)	0.76 (0.53-0.89)	-2.3; 1.3
Intra-rater with shoes	2.6 (±1.5)	0.4 (-0.1; 0.9)	0.65 (0.33–0.84)	-1.9; 2.6

### Part 2: concurrent validity

There were a significant correlation between ND measured with the stretch-sensor and ND measured with a ruler (r = 0.745, p < 0.001). The regression model showed a constant offset of 0.4 mm. (p < 0.001) and a correlation gradient of 1.0 (p = 0.084) indicating a systematic difference between the two methods. Mean ND measured with the stretch-sensor was 3.8 mm (±1.5) while mean ND measured with a ruler was 6.8 mm (±2.8).

## Discussion

This study showed that reliability of the stretch-sensor for measuring ND was acceptable when participants were walking without shoes (ICC 0.76–0.84). When participants were walking with shoes, the reliability decreased to ICC 0.65. ND measured with the stretch-sensor was significantly correlated with the static ND as performed by Brody. This suggests that the stretch-sensor has concurrent validity compared with the static ND. This new, simple method may hold promise for both clinical assessment and research as it enables reliable measurement of ND during walking in bare feet and while wearing shoes.

Intra- and inter-rater reliability of the stretch-sensor was within the same order of magnitude as the reliability demonstrated for static ND. The static ND test is often used to evaluate ND in a clinical setting, but the literature reports inconsistent results for its reliability. Vinicombe et al. found a moderate intra- and inter-rater reliability of the ND test of ICC 0.33–0.76 [21] while, Shultz et al. and Barton et al. both found ICC that were generally above 0.80 for both intra-rater reliability and higher inter-rater reliability [22,23]. This discrepancy of reliability suggests a need for a simple, consistent, and reliable method for a standard clinical ND measurement. The stretch-sensor displayed acceptable reliability for barefoot walking and the method is feasible for use in the clinic, as time-requirements are low. Reliability during shod walking was lower than what we hypothesized (ICC > 0.75) which suggests that ND during shod walking should be interpreted with care.

A significant association of r = 0.75 was found between the stretch-sensor and static ND as performed by Brody. This association suggests concurrent validity. However one of the reasons why this correlation is not higher could be because the two methods quantify two slightly different movement phenomena. Static ND measures the sagittal motion of the navicular tuberositas from neutral to loaded position. The stretch-sensor measures the change in distance between the medial malleoleus and the tuberositas navicular from the same neutral to loaded position. This change in distance measured by the stretch-sensor most likely combines ND in the sagittal plan and a medial drift of the navicular tuberosity in the transversal. Therefore ND measured by the stretch-sensor is likely a composite measure of midfoot mobility similar to the “Foot Mobility Magnitude” which also captures a single composite measure of midfoot movement [24]. This may question the relevance of the static ND as the appropriate measure to compare the stretch-sensor to. Future studies using 3D-motion capture to measure both drop and drift are needed before firm conclusion on the validity on the stretch-sensor can be made.

A challenge associated with mounting the stretch-sensor posterior to the navicular tuberosity can be skin movement artefacts. During the stance phase the ankle joint is positioned in 20 degrees dorsiflexion at heel strike which is immediately followed by plantar flexion, then moving from 0 to about 15 degrees dorsiflexion at heel-off [16]. During this phase skin movement artefact will likely influence the stretch-sensor at the tuberositas navicular. Based on the study by Wolf et al. [23] and Kappel et al. [15] we determined this position to be the most optimal while taking into account underlying bone movement and that the placement is feasible within shoes.

Even though the stretch sensor was found to have acceptable reliability, a limitation of the procedure is the manual registration of heel strike in the software DataAnalyzer. Training is needed to be able to detect heel strike in the software. The procedure is not time consuming, but it would be preferable to develop an algorithm for automatic registration, to avoid manual misinterpretations. This algorithm would help the clinician and researcher in the data analysis which would decrease time requirements.

Should ND be measured dynamically, or is it sufficient to measure static ND as performed by Brody to determine foot movement during daily activities? Rathleff et al. compared a static ND test with dynamic ND measured during gait with motion analysis. They found a very weak association between static and dynamic ND suggesting that a static test cannot be used to predict the dynamic ND [25]. 3D-motion analysis may be an option to measure dynamic ND. However, this laboratory-based method necessitates the need for special transparent shoes to measure in-shoe ND and prevent measurements being conducted while patients wear their own everyday shoes. These conclusions highlight the need for quick, simple, and dynamic measurements of ND, and the stretch-sensor seems suitable for such purposes but further studies are needed before the stretch-sensor can be recommended.

## Conclusions

This study suggests that the stretch-sensor has acceptable reliability for dynamic barefoot measurement of navicular drop. Furthermore, the stretch-sensor show concurrent validity compared with the static navicular drop test as performed by Brody. This new simple method may hold promise for both clinical assessment and research but more work is needed before the method can be recommended.

## Competing interests

MSR, OHS and SK were part of the group who invented the stretch-sensor used in the current study. The patent is owned by their employers, Aalborg University Hospital and Aarhus University and neither of the authors own any rights to the patent or technology. A patent application for the technology has been submitted under the application number PCT/DK2012/050341.

## Authors’ contributions

All authors participated in planning of the study. BHC, KSA, KSP and BSB were responsible for study design and data collection. All authors participated in data analysis and dissemination. BHC, KSA and MSR drafted the manuscript while KSP, BSB, OS and SLK all helped finalize the manuscript. All authors read and approved the final manuscript.
